# The Growing Demand for Peer Review: Current Challenges and Potential Reforms

**DOI:** 10.3389/bjbs.2025.14930

**Published:** 2025-07-14

**Authors:** Pierpaolo Pellicori, Jonathan Douxfils, Robert J. Mentz, John G. F. Cleland, Charlotte Beaudart

**Affiliations:** ^1^ School of Cardiovascular and Metabolic Health, University of Glasgow, Glasgow, United Kingdom; ^2^ Clinical Pharmacology and Toxicology Research Unit (URPC), Namur Research Institute for Life Sciences (NARILIS), Faculty of Medicine, University of Namur, Namur, Belgium; ^3^ QUALIblood sa, QUALIresearch, Liège, Belgium; ^4^ Department of Biological Hematology, CHU Clermont-Ferrand, Hôpital Estaing, Clermont-Ferrand, France; ^5^ Duke University Medical Center and Duke Clinical Research Institute, Durham, NC, United States; ^6^ Public Health Aging Research and Epidemiology (PHARE) Group, Clinical Pharmacology and Toxicology Research Unit (URPC), Namur Research Institute for Life Sciences (NARILIS), Faculty of Medicine, University of Namur, Namur, Belgium

**Keywords:** peer review, reviewers, ecosystem, system reform, academic publishing

## Introduction

“*I am sorry for declining on this occasion, but I receive too many review requests and cannot accept all of them. Researchers cannot continue to work for free. The peer review ecosystem needs to change.*”

I, Dr Pellicori, was initially surprised to read this comment from Charlotte when she declined to review a manuscript that I was handling. However, I also felt that there might be deeper reasons behind her strong response, ones that I have often wished to voice to an editor myself. At times, I am also an overwhelmed reviewer.

## The Importance and Challenges of the Peer-Review Process

Peer review has been central to academic publishing for over a century and continues to evolve [1]. Researchers are well acquainted with the process, which is designed to support the integrity of scientific research, by providing expert constructive feedback that enhances the rigour and quality of academic work. This practice is very important, as it aids editors in determining which articles merit publication, thereby improving the quality of research disseminated to the wider scientific community.

With a growing volume of submissions and the proliferation of medical journals, many academics are asked to provide several reviews every week, often with tight deadlines. Although reviewing has educational benefits other merits and professional rewards, peer review remains a voluntary, unpaid task that requires considerable time and intellectual effort, and expert knowledge of the scientific literature [2, 3]. Overall, reviewers contribute >100 million hours every year to peer review [4]. The majority of academics produce reviews in the evenings or at weekends, in addition to managing their own heavy workloads, and their family and social lives. This can lead to prolonged turnaround times and incomplete assessments, diverting attention from other important tasks. Reviewer fatigue compromises the quality of feedback and may lead to failure to identify critical flaws in a paper. Worryingly, more than 10,000 retractions were reported in 2023 alone [5]. This is a substantial waste of resources. The problem is further compounded by an over-reliance on a small pool of academics who are more likely to respond to and accept requests, which intensifies the burden on these cooperative volunteers. There is no doubt that the majority of reviewers are becoming much more selective, only responding to review requests from editors they are familiar with, journals they are associated with, or those that they would consider publishing in.

*“If we all stopped agreeing to review for free who would review our own manuscripts? Rather than resisting and risking the collapse of the system, perhaps we should focus on proposing more constructive solutions … but what would those look like?*” [SIC]

## An Evolution of the Peer-Review Ecosystem Is Needed

To meet the demands of modern research, the peer-review ecosystem must evolve, incentivising reviewer participation, enhancing transparency, and enforcing better standards for data verification and methodology, while at the same time allowing authors to express ideas that challenge convention.

Different models of peer review have been trialled and implemented in recent years. While most journals keep reviewers’ identities anonymous to authors, some are shifting towards open peer review, in which reviewers’ identities are disclosed alongside their evaluations. This approach may introduce greater transparency, but it limits what the reviewer is willing to write. Adverse open reviews may cause disputes among colleagues. Open review may create bias, either because legitimate criticism is withheld to avoid causing offence or because of inappropriately positive reviews in the hope of benefits in terms of career progression or a reciprocal favour when the reviewer submits their next paper. The potential for collusion with open review is substantial.

Typically, each manuscript is evaluated by two or more independent reviewers. Adopting a pre- and post-publication review model that encourages multiple reviewers to engage in discussions on social media platforms could accelerate the dissemination of research and provide ongoing feedback. However, open participation may also lead to subjective or uninformed comments and potential discreditation. Many other models are currently being explored [6]; incorporating technological innovations, including artificial intelligence, could help to create a more modern, efficient - and sustainable - peer-review ecosystem [7].

One of the main concerns we repeatedly hear from our academic colleagues is the lack of formal recognition or reward for their substantial contributions to peer review. While listing the hundreds of manuscripts and grants reviewed on platforms such as ORCID or Web of Science may showcase extensive contributions to the community, this still adds little value to academic careers. In response, some journals have introduced non-financial incentives such as discounts on publication fees, free access to articles in the journal, continuing medical education credits, or public acknowledgement.

What if financial rewards were introduced, especially given that researchers are now being pushed to pay thousands of dollars to have their manuscripts peer-reviewed and published open-access? The current system disproportionately benefits publishers from a financial standpoint. Redirecting a portion of these funds back to individual researchers or – why not - universities would establish a more equitable model. This shift could be vital not only for the current peer-review system but also for the academic community, as it could help to retain early-career researchers and enhance job security, stability and satisfaction. It could also provide an additional metric to support career advancement.

To “quantify” reviewer activities, it may be necessary to establish a global platform that provides tools to evaluate the quality and relevance of reviews based on factors such as quality, timeliness and impact. This platform would also offer publicly accessible metrics. Editors would play a central role in evaluating reviews, rating them for thoroughness and actionable insights. In other words, this would create a system that incentivises and enhances the quality of reviews more fairly and sustainably. We believe the publishing industry has the resources and a sense of duty to support these initiatives, although the appointment of more editors would be required to fulfil this task. We should not forget that many journal editors receive only a modest stipend or work voluntarily.

Educational activities aimed at nurturing the next-generation of reviewers and editors could be both constructive and fulfilling. For example, a few years ago, the Journal of Cardiac Failure launched a Reviewer Mentorship Programme, which involved pairing junior mentees with senior mentors to guide them through the review process and provide direct feedback. The programme also included monthly didactic sessions and opportunities for participants to attend editorial board meetings. Outstanding reviewers were recognised with promotions to the editorial board or opportunities to write editorials [8, 9]. Many scientific organisations and publishers are creating their own educational initiatives [10].

## Discussion

Reviewers, editors, and publishers bear great responsibility. Rigorous research and high-quality peer reviews are both essential for pushing the boundaries of knowledge and advancing public health on a global scale, providing the evidence needed for decisions that impact billions of lives. Recognising the time and expertise invested in peer review and editorial work is essential for sustaining the academic publishing ecosystem.

## Data Availability

The original contributions presented in the study are included in the article/supplementary material, further inquiries can be directed to the corresponding author.
